# P2X_3_ Receptors Mediate Visceral Hypersensitivity during Acute Chemically-Induced Colitis and in the Post-Inflammatory Phase via Different Mechanisms of Sensitization

**DOI:** 10.1371/journal.pone.0123810

**Published:** 2015-04-17

**Authors:** Annemie Deiteren, Laura van der Linden, Anouk de Wit, Hannah Ceuleers, Roeland Buckinx, Jean-Pierre Timmermans, Tom G. Moreels, Paul A. Pelckmans, Joris G. De Man, Benedicte Y. De Winter

**Affiliations:** 1 Laboratory of Experimental Medicine and Pediatrics, Division of Gastroenterology, University of Antwerp, Antwerp, Belgium; 2 Laboratory of Cell Biology and Histology, University of Antwerp, Antwerp, Belgium; 3 Antwerp University Hospital, Department of Gastroenterology and Hepatology, Antwerp, Belgium; University of California, Los Angeles, UNITED STATES

## Abstract

**Objectives:**

Experiments using P2X_3_ knock-out mice or more general P2X receptor antagonists suggest that P2X_3_ receptors contribute to visceral hypersensitivity. We aimed to investigate the effect of the selective P2X_3_ antagonist A-317491 on visceral sensitivity under physiological conditions, during acute colitis and in the post-inflammatory phase of colitis.

**Methods:**

Trinitrobenzene sulphonic-acid colitis was monitored by colonoscopy: on day 3 to confirm the presence of colitis and then every 4 days, starting from day 10, to monitor convalescence and determine the exact timepoint of endoscopic healing in each rat. Visceral sensitivity was assessed by quantifying visceromotor responses to colorectal distension in controls, rats with acute colitis and post-colitis rats. A-317491 was administered 30 min prior to visceral sensitivity testing. Expression of P2X_3_ receptors (RT-PCR and immunohistochemistry) and the intracellular signalling molecules cdk5, csk and CASK (RT-PCR) were quantified in colonic tissue and dorsal root ganglia. ATP release in response to colorectal distension was measured by luminiscence.

**Results:**

Rats with acute TNBS-colitis displayed significant visceral hypersensitivity that was dose-dependently, but not fully, reversed by A-317491. Hypersenstivity was accompanied by an increased colonic release of ATP. Post-colitis rats also displayed visceral hypersensitivity that was dose-dependently reduced and fully normalized by A-317491 without increased release of ATP. A-317491 did not modify visceral sensitivity in controls. P2X_3_ mRNA and protein expression in the colon and dorsal root ganglia were similar in control, acute colitis and post-colitis groups, while colonic mRNA expression of cdk5, csk and CASK was increased in the post-colitis group only.

**Conclusions:**

These findings indicate that P2X_3_ receptors are not involved in sensory signaling under physiological conditions whereas they modulate visceral hypersensitivity during acute TNBS-colitis and even more so in the post-inflammatory phase, albeit via different mechanisms of sensitization, validating P2X_3_ receptors as potential new targets in the treatment of abdominal pain syndromes.

## Introduction

Abdominal pain is a common symptom in many gastrointestinal disorders, among which the irritable bowel syndrome (IBS) and inflammatory bowel disease (IBD). In IBD, the continuous release of inflammatory mediators during flares can sensitize peripheral nerve endings in the gut wall, resulting in disturbed sensitivity and abdominal pain, which is not only present during acute flares of inflammation but also during remission [1,2]. In contrast in IBS, abdominal pain and visceral hypersensitivity persist in the absence of overt colonic pathology [3]. Of note, a specific subgroup of patients develops IBS after an episode of acute gastroenteritis, termed post-infectious IBS [4]. Current treatments for IBD and IBS are mainly aimed at dampening the intestinal inflammation (IBD) or normalizing the associated motility dysfunctions (IBS) to indirectly reduce abdominal pain; in contrast, few therapeutics specifically target the afferent nerves.

Extracellular adenosine 5’-triphospate (ATP) has been established as a key sensory signaling molecule that activates purinergic P2X ligand-gated ion channel receptors and P2Y G-protein coupled receptors [5]. There are seven P2X receptor subunits (P2X_1-7_) that can be assembled as homo- or heteromultimers [6]. Especially P2X_3_ receptor units, expressed as homomeric P2X_3_ or heteromeric P2X_2/3_ receptors, are considered to play a major role in visceral sensory function and have been put forward as an interesting target in the pursuit of new treatments for visceral pain such as in IBS and IBD [7,8]. ATP, released from the epithelium lining cells upon distension of hollow organs (e.g. gut, urinary and gall bladder and lung), acts on these P2X_3_ channels which are expressed on subepithelial nerves relaying the sensory information to the central nervous system [5]. In addition, P2X_3_ receptors are the predominant purinergic receptor subtypes present in the dorsal root ganglia (DRGs) [9,10]. They are found mainly on small and medium-sized neurons, which are considered to be nociceptive C-fibers, but also on a subpopulation of Aδ-fibers [9,11,12]. Considering the predominant expression on nociceptive afferent fibers, the role of purinergic signaling in visceral pain and hypersensitivity has been intensively studied. However, so far the functional evidence implicating the P2X_3_ and P2X_2/3_ receptor subtypes in gut sensory signaling has come mainly from experiments using either knock-out mice or nonselective P2X antagonists such as TNP-ATP, that acts on P2X_3_ and P2X_2/3_ receptors but also displays nanomolar affinity for P2X_1_, or PPADS that blocks all types of P2X receptors [13–20]. In contrast, the potential of specific P2X_3_ unit targeted therapy has been hardly explored in animal models of gut hypersensitivity [20]. Nonetheless, such antagonists have already progressed to phase II clinical trials for hypersensitivity-associated disorders such as interstitial cystitis, osteoarthritis and idiopathic chronic cough (http://clinicaltrials.gov) [11]. In addition, most studies have been conducted under physiological conditions or in animal models of acute chemically-induced visceral hypersensitivity [14–17,19,20]. Despite its relevance from a clinical point-of-view, little *in vivo* evidence is available on the role of P2X_3_ receptor modulation of visceral sensory function in models of longer-lasting visceral pain.

Therefore, in this study we investigated the effect of A-317491, a selective and potent antagonist of P2X_3_ channels and evaluated its antinociceptive potential under physiological conditions, during acute colitis and in a model for post-inflammatory visceral hypersensitivity. We demonstrated that A-317491 dose-dependently reduced visceral hypersensitivity with a different potency during acute colitis and in the post-inflammatory phase, without affecting visceral nociception under physiological conditions.

## Material and Methods

### Animals

Male Sprague-Dawley rats (200–225 g) were obtained from Charles River. Animals were kept in pairs at a constant room temperature (22 ± 2°C) and humidity (60%) and on a 12h:12h day-night cycle. They were allowed to acclimatize to housing conditions for 1 week prior to experimentation. All animal procedures were approved by the Ethical Committee for use of Experimental Animals at the University of Antwerp (2010–18). Except for the assessment of visceral sensitivity which requires rats to be fully awake, all procedures were performed under pentobarbital anesthesia (45–100 mg/kg). All efforts were made to minimize animal suffering.

### Induction of colitis

TNBS-colitis was induced after an overnight fast and under pentobarbital anesthesia (60 mg/kg) by intrarectal administration of a 0.5 ml enema containing 15 mg of TNBS, dissolved in 50% ethanol [21,22]. Control rats were administered an intrarectal saline enema.

### *In vivo* markers of inflammation

Colonoscopy was performed to confirm the extent of colitis and to monitor convalescence longitudinally in time as previously described [21]. The lubricated tip of a baby gastroscope (Olympus Europa GmbH) was inserted through the anus of the sedated rat and advanced under endoscopic control for 10 cm. During withdrawal, TNBS-induced mucosal damage was assessed using a standardized scoring system, taking into account the degree of ulceration, disease extent and the presence of edema, bleeding or stenosis (total score 0–19) [23].

### Post-mortem markers of inflammation

At the end of the experiments, the distal colon was rapidly excised to evaluate macroscopic mucosal damage using a validated scoring system (score 0–10) [23,24]. A representative section was fixed in 4% formaldehyde for 24 h and embedded in paraffin for hematoxylin-eosin staining. Histological specimens (5 μm) were scored microscopically for the presence of an inflammatory infiltrate, the number of infiltrated layers, mucosal architectural distortion and edema (score 0–10) [21,23]. A second representative 1 cm segment was used to assay myeloperoxidase (MPO) activity according to our previously published methods [23]. In brief, specimens were blotted dry and placed in a potassium phosphate buffer pH 6.0 containing 0.5% hexadecyltrimethylammonium bromide (5 g per 100 ml buffer). The samples were arranged on ice, homogenized for 30 s and subjected to two sonication and freeze-thawing cycles. The suspension was centrifuged at 15000 g for 15 min at 4°C. Aliquots (100 μl) of the supernatant were added to 2.9 ml of *o*-dianisidine solution (16.7 mg of *o*-dianisidine in 1 ml saline, 98 ml of 50 mmol potassium phosphate buffer, pH 6.0 and 1 ml of a 0.05% H_2_O_2_ solution as a substrate for MPO enzyme). The change in absorbance was read at 460 nm over 60 s using a Spectronic Genesys 5 spectrophotometer (Milton Roy). One unit of MPO activity was defined as the quantity able to convert 1 μmol H_2_O_2_ to H_2_O per min at 25°C and was expressed as units per gram of tissue (U/g tissue).

### Visceral sensitivity

Visceral sensitivity was assessed by quantifying the VMRs to colorectal distension [25,26]. The VMR is a nociceptive reflex that integrates both peripheral and central mechanisms and consists of the contraction of the abdominal musculature in response to noxious colorectal distension. Three to five days prior to VMR assessment, two Teflon-coated EMG electrodes were sutured into the external oblique abdominal muscle and exteriorized at the base of the neck for future access. On the day of visceral sensitivity testing, a lubricated balloon (5 cm length) was carefully inserted in the colorectum up to 0.5 cm passed the anal verge. The balloon catheter was secured to the tail and connected to a barostat system (Distender Series II Barostat, G&J Electronics) for pressure-controlled, graded colorectal distensions (10–80 mmHg, 20 s, 4 min interval). The EMG electrodes were relayed to a data acquisition system and the abdominal EMG signal was recorded (NL100AK headstage), amplified (NL104), filtered (NL 125/126, Neurolog, Digitimer Ltd, bandpass 50–5000 Hz) and digitized (CED 1401, Cambridge Electronic Design,) to a PC for off-line analysis using Spike2 version 5.16 for Windows (Cambridge Electronic Design). The analog EMG signal was rectified and integrated. To quantify the magnitude of the VMR at each distension pressure, the area under the curve (AUC) during distension (20 s) was corrected for the baseline activity (AUC pre-distension, 20 s).

### Colonic compliance

Colonic compliance was studied in the same animal to exclude pharmacologically mediated changes in the viscoelastic properties of the colonic wall as the mechanism of antinociception. Rats were anesthetized (pentobarbital 45 mg/kg) and graded volumes of saline (0–2.5 ml) were applied to the balloon inserted in the colorectum while recording the corresponding intracolonic pressure.

### ATP assay

ATP release was assayed as previously published [16,17]. Under deep pentobarbital anesthesia (100 mg/kg) and after intrathoracic exsanguination, the distal colon was rapidly excised and transferred to an organ bad, which was continuously perfused with oxygenated Krebs solution (118 mM NaCl, 4.75 mM KCl, 1 mM NaH_2_PO_4,_ 22 mM NaHCO_3_, 1.2 mM MgSO_4_, 2.5 mM CaCl_2_, 11 mM D-glucose; 10 ml/min). The proximal and distal ends of the colon were secured to an in- and outflow port respectively for intraluminal perfusion with Krebs solution that was maintained at a constant rate of 1 ml/min via a perfusion syringe pump. Colorectal distension was induced by closure of the outflow while maintaining the inflow, resulting in ramp distension (up to 60 mmHg). A pressure transducer was placed in parallel to monitor intracolonic pressure. The distension fluid was collected, snap frozen and stored at -80°C. The concentration of ATP in the samples was quantified using a luciferin-luciferase ATP determination assay (A22066; Invitrogen).

### Quantitative RT-PCR

Expression of P2X_3_ receptors and of cdk5, csk and CAS, molecular determinants of P2X_3_-mediated signaling, were quantified in the colon and dorsal root ganglia (DRGs). Colonic segments were harvested from sites of representative macroscopic appearance. The DRGs contain the primary afferent neurons conveying sensory information from the colon to the spinal cord and were harvested bilaterally at Th12-L2 and at L6-S1. Total RNA was extracted from colon using the RNeasy Minikit (Qiagen) and from DRGs using the Absolutely RNA microprep kit (Stratagene). RNA was then converted to cDNA (Transcriptor First Strand cDNA Synthese Kit, Roche). A Taqman gene expression assay (Applied Biosystems) was performed for P2X_3_ (Rn00579301_m1), cdk5 (Rn04219635_m1), csk (Rn01418228_m1) and CASK (Rn00573365_m1) on a ABIPrism 7300 sequent detector system (Applied Biosystems) in a 25 μl reaction volume containing 2 μl cDNA, 12.5 μl TaqMan Universal PCR master mix (Applied Biosystems), 1.25 μl Taqman assay probe and 9.25 μl RNase-free H_2_O. The parameters for PCR amplification were 50°C for 2 min, 95°C for 10 min, followed by 40 cycles of 95°C for 15 s and 60°C for 1 min. Expression was normalized against the reference gene β-actin (Rn00667869_m1; Applied Biosystems; stable across tissues and experimental conditions) for calculation of comparative cycle thresholds [ΔC_T_ = C_T_(target gene)—C_T_(β-actin)]. Relative expression of mRNA species was then determined as 2^-ΔΔCT^ with ΔΔCT = ΔC_T_ (TNBS)- ΔC_T_ (control) [27].

### Immunohistochemistry

Animals were transcardially perfused with 4% paraformaldehyde in phosphate buffer (pH 7.4). The DRGs (Th13-L2 to L6-S1) were dissected out and post-fixed for 30 min. After PBS washing, DRGs were incubated in 0.01M PBS with 20% sucrose at 4°C overnight and embedded in OCT medium for cryopreservation. Cryosections (9 μm) were thaw-mounted on poly-L-lysine-coated microscope slides. As a blocking step, cryosections were incubated for 1 h with 0.01M PBS (pH 7.4) containing 0.05% thimerosal (PBS*) supplemented with 10% normal horse serum (NHS) and 1% Triton X-100. Goat polyclonal anti-CGRP (calcitonin gene-related peptide; AB36001, 1:2500; Abcam) and rabbit polyclonal anti-P2X_3_ (AB5895, 1:500; Millipore) were diluted in PBS* supplemented with 10% NHS and applied overnight at room temperature. After rinsing in PBS, tissues were incubated with Cy3-labeled donkey anti-goat (1:4000) and FITC-labeled donkey anti-rabbit (1:300; both Jackson Immunoresearch) diluted in 0.01M PBS (pH 7.4) and again rinsed in PBS. Cryosections were mounted in citifluor (Citifluor Ltd) and visualized with fluorescence microscopy. For quantification, 3 thoracolumbal (Th13-L2) and 3 lumbosacral (L6-S1) DRGs were evaluated per animal (n = 3 animals per experimental condition: control, acute colitis, post-colitis). Three non-sequential cryosections were counted per DRG for expression of CGRP, P2X_3_ and co-expression levels, as these were previously reported to be enhanced in another animal model for visceral hypersensitivity [17].

### Experimental protocol

**Fig 1:**
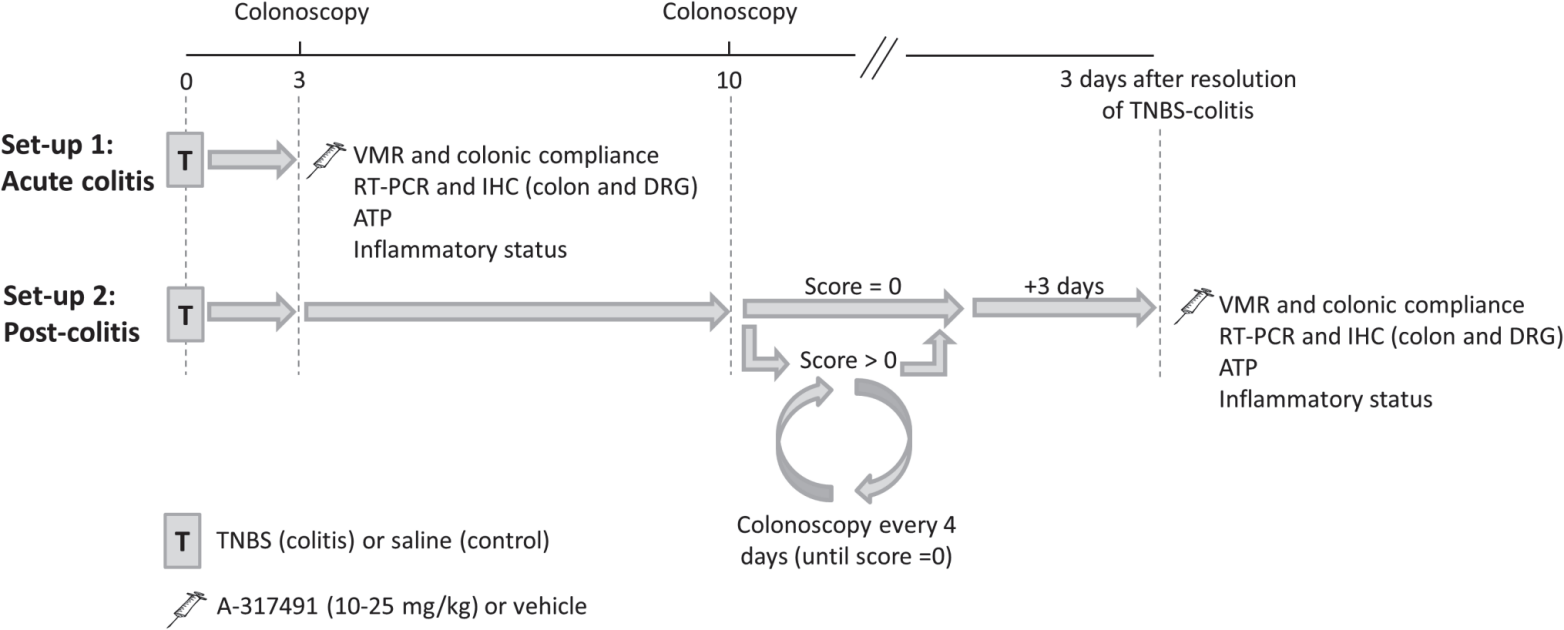
Scheme of the two experimental set-ups. In set-up 1 (acute colitis), rats were instilled with TNBS (colitis) or saline (control) and further experiments were conducted 3 days later, during the acute phase of colitis. In set-up 2 (post-colitis), rats were instilled with TNBS or saline and the extent of colitis and the healing process were monitored individually by repeated colonoscopy: first on day 3 to confirm the presence of colitis and thereafter, starting from day 10, every 4 days until complete mucosal healing (score = 0) occurred. Further experiments were conducted 3 days after colonoscopically proven mucosal healing. A-317491 (10–25 mg/kg) or vehicle (saline), denoted by the injection needle, was administered 30 min prior to the start of the VMR protocol. Evaluation of the inflammatory status entailed colonoscopy, macroscopic and microscopic assessment of the colonic tissue in addition to a myeloperoxidase activity (MPO) assay. ATP, adenosine 5’-triphosphate; DRG, dorsal root ganglion; IHC, immunohistochemistry; RT-PCR, reverse transcription polymerase chain reaction; TNBS, trinitrobenzene sulphonic acid; VMR, visceromotor response.

Experiments were performed either during acute colitis or in the post-colitis phase (Fig 1). In set-up 1, the role of P2X_3_ receptors in sensory signaling was investigated during acute colitis. Rats were randomized to receive a saline (control) or TNBS enema and all experiments were conducted 3 days later, during acute inflammation. In set-up 2, the contribution of P2X_3_ to visceral hypersensitivity was studied in the post-inflammatory phase of colitis. Rats were randomized to receive a saline (control) or TNBS (colitis) enema and the extent of inflammation was verified colonoscopically on day 3. From day 10 onwards, convalescence was monitored individually by repeated colonoscopy which was performed every 4 days. If at any time point colonoscopy still showed signs of mucosal inflammation, the animal was allowed to recover further and colonoscopy was repeated 4 days later. If colonoscopy showed complete mucosal healing, all experiments were conducted 3 days later.

In both the acute and post-colitis set-ups, rats received a single injection with A-317491, a selective P2X_3_ receptor antagonist (10–25 mg/kg ip) or vehicle (saline ip) 30 min prior VMR assessment, which was followed immediately by an evaluation of colonic compliance. In each rat, colonoscopy, as well as macroscopic and microscopic evaluation of the colonic tissue were performed in addition to an MPO activity assay to quantify inflammatory markers. To assess P2X_3_ receptor expression at the mRNA and protein level and quantify the expression of the intracellular signaling molecules of P2X_3_ signaling, the colon and DRGs were harvested from drug-naive rats in the control, acute colitis and post-colitis groups. In addition, colorectal distension was performed *in vitro* and the distension fluid was collected to assay ATP release.

### Statistical analysis

Data are presented as mean ± sem for n the number of animals per group. Variables were analyzed using unpaired Student’s *t*-test and one-way or two-way ANOVA followed by Student-Newman-Keuls (SNK) post-hoc test when appropriate. Analysis of VMR and compliance data was performed by the generalized estimating equation (GEE) model followed by least significant difference (LSD) post-hoc test when appropriate. Statistical analysis was executed using SPSS 20.0 software. Statistical significance was set at p<0.05.

## Results

### Effect of P2X_3_ receptor blockade on visceral hypersensitivity during acute TNBS-colitis

Three days after TNBS-instillation distal colitis was present, characterized by the presence of multiple serpiginous ulcers, whereas no colonic damage was seen in controls. Inflammatory markers were significantly increased in TNBS-instilled rats (Table 1).

**Table 1:** Inflammatory status during acute TNBS-colitis.

Group	Drug	N	Colonoscopy	Macroscopy	Microscopy	MPO
			(0–19)	(0–10)	(0–10)	(U/g)
Control	Vehicle	n = 8	0.0 ± 0.0	0.0 ± 0.0	0.0 ± 0.0	0.4 ± 0.1
	25 mg/kg A-317491	n = 5	0.0 ± 0.0	0.0 ± 0.0	0.0 ± 0.0	1.0 ± 0.6
Acute colitis	Vehicle	n = 8	7.0 ± 0.9 ***	6.3 ± 0.8 ***	4.4 ± 0.7 ***	16.5 ± 0.7 **
	10 mg/kg A-317491	n = 8	7.3 ± 0.9 ***	6.0 ± 0.7 ***	4.6 ± 0.8 ***	27.7 ± 0.6 **
	25 mg/kg A-317491	n = 8	6.5 ± 0.5 ***	4.4 ± 1.2 ***	3.2 ± 1.2 ***	14.9 ± 3.5 **

Results are presented as mean ± sem. Two-way ANOVA followed by SNK post-hoc test

** p<0.01

*** p<0.001, significant effect of the factor colitis; no significant effect of the factor drug; no interaction.

**Fig 2:**
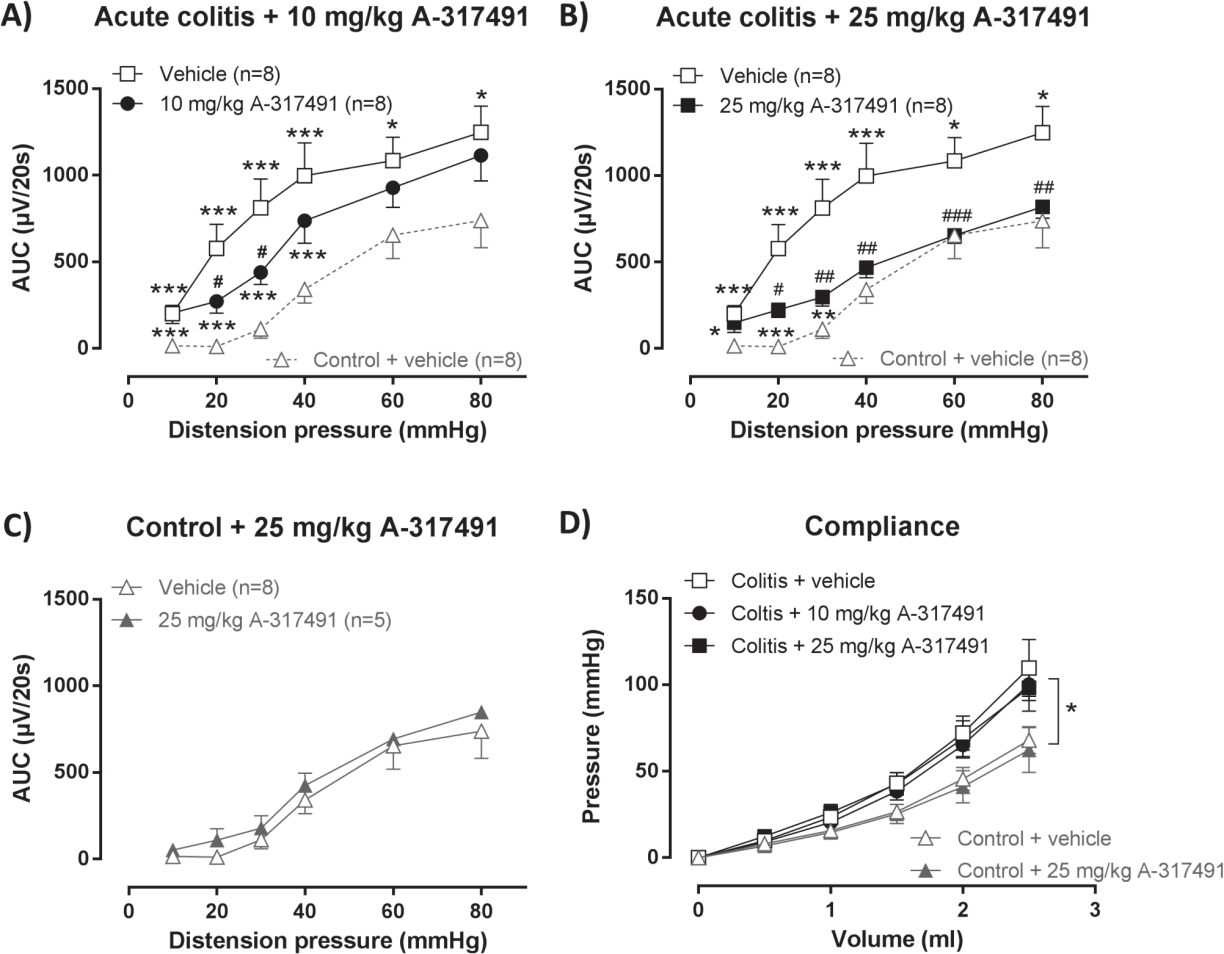
Effect of A-314791 on VMRs and colonic compliance during acute TNBS-colitis. VMRs were assessed 3 days post-TNBS enema, immediately followed by an evaluation of colonic compliance. A-314791 (filled symbols; 10–25 mg/kg) or vehicle (open symbols) was administered 30 min prior to VMR assessment. Increased VMRs were present in rats with acute TNBS-colitis compared to controls and were dose-dependently reduced by 10 mg/kg (**A**) and 25 mg/kg (**B**) A-317491. The 25 mg/kg dose did not affect VMRs in healthy controls (**C**). To facilitate comparison, VMRs for vehicle-treated control rats are also shown in A and B (gray dashed line). Generalized estimating equations, LSD post-hoc test, n = 5–8; * p<0.05, ** p<0.01, *** p<0.001, significantly different from control + vehicle; ^#^ p<0.05, ^##^ p<0.01, ^###^ p<0.001, significantly different from acute colitis + vehicle. Colonic compliance was reduced during acute TNBS-colitis, but remained unaffected by A-317491 (10–25 mg/kg) (**D**). Generalized estimating equations, LSD post-hoc test, n = 5–8; * p<0.05, significant effect of the factor colitis.

VMRs to colorectal distension were markedly increased during acute TNBS-colitis compared to controls, indicating the presence of acute inflammation-induced visceral hypersensitivity (Fig 2A). This hypersensitivity was dose-dependently reduced by A-317491. After 10 mg/kg of A-317491 VMRs were not significantly reduced at the highest distension pressures of 40–80 mmHg but were markedly attenuated at the lower distension pressures (20 and 30 mmHg). In contrast, 25 mg/kg significantly reduced hypersensitivity for almost the full range of distension pressures (20 to 80 mmHg) (Fig 2B). However, VMRs remained significantly increased compared to controls at 10 to 30 mmHg distension. The 25 mg/kg dose did not affect VMRs in the control group (Fig 2C).

Colonic compliance was assessed by distending the balloon in the colorectum with increasing volumes of water which increased the intracolonic pressure in a volume-dependent fashion (Fig 2D). Intracolonic pressures increased more rapidly during acute colitis, indicating reduced colonic compliance compared to controls. The pressure-volume relationship was not modulated by either dose of A-317491. In addition, the single administration of A-317491 30 min before assessment of the VMR did not affect the colonoscopic, macroscopic or microscopic appearance of the colonic tissue (Table 1).

### Effect of P2X_3_ receptor blockade on post-inflammatory visceral hypersensitivity

TNBS-instillation resulted in acute colitis, as evidenced by the colonoscopic scores on day 3 (Table 2). Colitis resolved spontaneously after a median of 14 days (range 10–14 days). Analysis of the inflammatory markers confirmed the post-inflammatory status at the time of the experiment (Table 2).

**Table 2:** Inflammatory status after the resolution of TNBS-colitis.

Group	Drug	N	Colonoscopy (0–19)		Macroscopy	Microscopy	MPO
			day 3	after exp	(0–10)	(0–10)	(U/g)
Control	Vehicle	n = 8	0.0 ± 0.0	0.0 ± 0.0	0.0 ± 0.0	0.0 ± 0.0	0.9 ± 0.4
	25 mg/kg A-317491	n = 5	0.0 ± 0.0	0.0 ± 0.0	0.0 ± 0.0	0.0 ± 0.0	1.0 ± 0.3
Post-colitis	Vehicle	n = 8	5.2 ± 0.6 ***	0.3 ± 0.3	0.3 ± 0.3	0.3 ± 0.3	0.6 ± 0.2
	10 mg/kg A-317491	n = 8	5.2 ± 0.6 ***	0.0 ± 0.0	0.3 ± 0.3	0.0 ± 0.0	1.2 ± 0.3
	25 mg/kg A-317491	n = 7	5.3 ± 0.6 ***	0.0 ± 0.0	0.0 ± 0.0	0.0 ± 0.0	0.6 ± 0.2

Results are presented as mean ± sem. Two-way ANOVA followed by SNK post-hoc test

*** p<0.001, significant effect of the factor colitis; no significant effect of the factor drug; no interaction. Exp, experiment.

**Fig 3:**
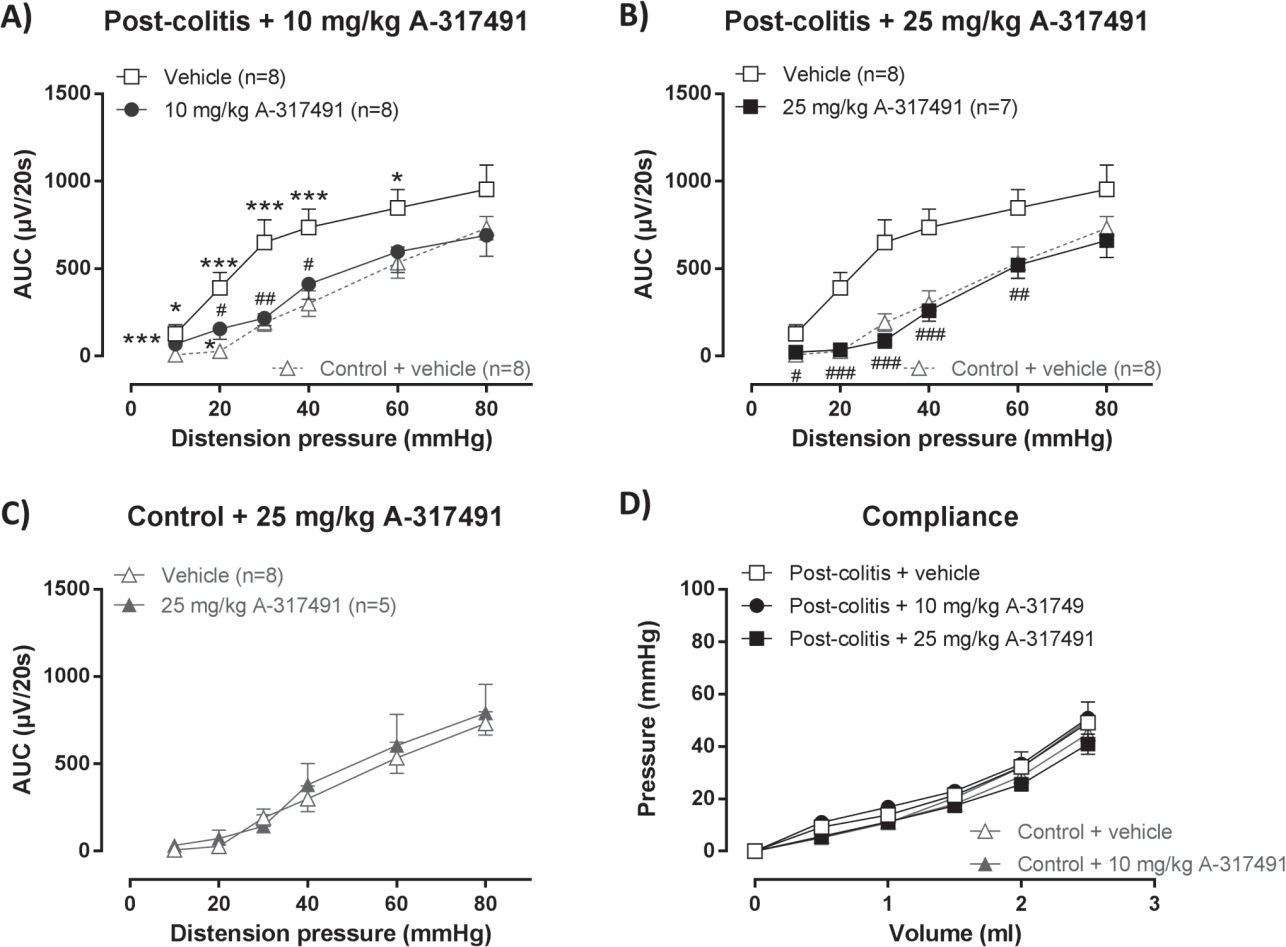
Effect of A-314791 on VMRs and colonic compliance after the resolution of TNBS-colitis. VMRs were assessed 3 days after colonoscopically-proven resolution of TNBS-colitis, immediately followed by an evaluation of colonic compliance. A-314791 (filled symbols; 10–25 mg/kg) or vehicle (open symbols) was administered 30 min prior to VMR assessment. Increased VMRs were present in rats that had recovered from colitis compared to controls and were normalized by 10 mg/kg (**A**) and 25 mg/kg (**B**) A-317491. The 25 mg/kg dose did not affect VMRs in healthy controls (**C**). To facilitate comparison, VMRs for vehicle-treated control rats are also shown in A and B (gray dashed line). Generalized estimating equations, LSD post-hoc test, n = 5–8; * p<0.05, *** p<0.001, significantly different from control + vehicle; ^#^ p<0.05, ^##^ p<0.01, ^###^ p<0.001, significantly different from acute colitis + vehicle. Colonic compliance was similar in control and post-colitis rats and remained unaffected by A-317491 (10–25 mg/kg) (**D**). Generalized estimating equations, LSD post-hoc test, n = 5–8.

After resolution of TNBS-colitis, VMRs were significantly increased in post-colitis rats compared to controls for the full range of distension pressures, indicating the presence of marked post-inflammatory visceral hypersensitivity (Fig 3A). These increased VMRs were reduced by A-317491. After a single administration of 10 mg/kg A-317491, VMRs were markedly attenuated (Fig 3A). However VMRs remained significantly increased compared to controls at 20 mmHg distension. After treatment with 25 mg/kg A-317491, VMRs normalized over the full range of distension pressures and were no longer significantly different from those of controls at any of the distension pressures applied (Fig 3B). The highest dose of 25 mg/kg did not affect VMRs in control rats (Fig 3C).

After resolution of TNBS-colitis, colonic compliance was similar in the control and post-colitis rats and was not altered by either dose of A-317491 (Fig 3D). In addition, A-317491 treatment had no effect on inflammatory markers (Table 2).

### Effect of P2X_3_ receptor blockade in acute inflammatory compared to post-inflammatory visceral hypersensitivity

**Fig 4:**
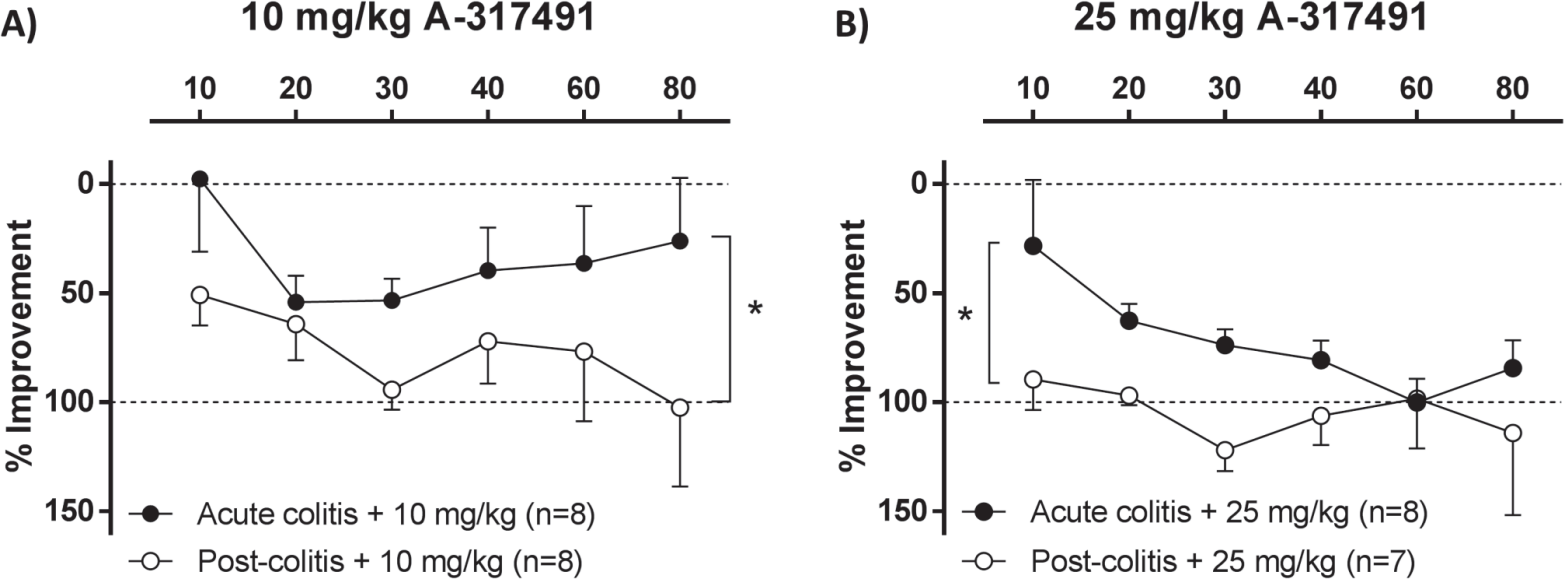
Effect of A-317491 in acute TNBS-colitis compared to the post-inflammatory phase of colitis. To substantiate a difference in potency of A-317491 to reduce visceral hypersensitivity during acute TNBS-colitis compared to the post-inflammatory phase of colitis, data are expressed as the percentage of improvement of normalization: 0% means no improvement and thus the same level of hypersensitivity as vehicle-treated rats during acute TNBS-colitis or in the post-inflammatory phase, whereas 100% means complete normalization of the increased VMRs (reaching the level of vehicle-treated controls). Both the 10 mg/kg (**A**) and the 25 mg/kg (**B**) dose of A-317491 more potently reduced visceral hypersensitivity in the post-inflammatory phase of colitis compared to the acute inflammatory phase of colitis. Generalized estimating equations, LSD post-hoc test, n = 7–8; * p<0.05, significantly different compared to acute colitis.

During acute colitis, A-317491 dose-dependently, but not fully reversed visceral hypersensitivity, even at the highest dose of 25 mg/kg (Fig 2). However, in post-colitis rats the 10 mg/kg dose already normalized VMRs (Fig 3). To substantiate a difference in potency of A-317491 in reducing visceral hypersensitivity between the acute phase of colitis and the post-inflammatory phase, the effect of A-317491 on VMRs was expressed as the percentage of improvement or normalization (Fig 4): 0% indicates no improvement (compared to vehicle-treated acute colitis or post-colitis rats) whereas 100% accounts for full normalization of the VMRs (to the level of vehicle-treated controls). In post-colitis rats both the 10 and the 25 mg/kg dose of A-317491 were more effective in reducing the increased VMRs compared to the effect of A-317491 in rats in the acute phase of colitis (Fig 4).

ATP release in response to 60 mmHg of colorectal distension was significantly increased in the distal colon of rats with acute TNBS-colitis (20.0 ± 6.4 pmol/ml; n = 11; p<0.05) compared to controls (3.4 ± 2.2 pmol/ml; n = 8). In the post-colitis condition, ATP release was no longer significantly different compared to controls (2.6 ± 0.9 pmol/l; n = 6).

**Fig 5:**
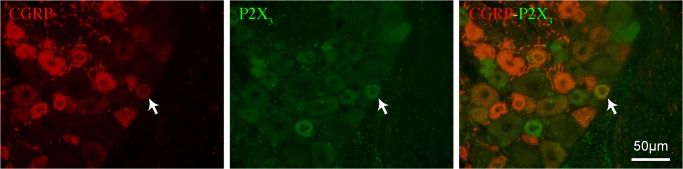
Expression of CGRP and P2X_3_ in dorsal root ganglia. Immunohistochemical detection of CGRP (left panel) and P2X_3_ (middle panel) on cryosections of a dorsal root ganglion. The right panel shows the merged picture; the white arrows indicate neuronal co-expression of CGRP and P2X_3_.

The relative expression of P2X_3_ unit mRNA in the colon and DRGs was comparable in control, acute colitis and post-colitis rats (Table 3). Immunohistochemical staining of DRGs also revealed no difference in expression of CGRP and P2X_3_ between control, acute colitis and post-colitis conditions (Table 4; Fig 5). In addition, co-expression levels were similar between all groups. However, RT-PCR did reveal a difference in the mRNA expression of cdk5, csk and CASK, important molecular determinants of P2X_3_ receptor-mediated signaling (Table 3). Colonic mRNA expression of all three targets was increased in the post-colitis group, whereas mRNA levels were similar in the colon of acute colitis and control rats. The increased expression in post-colitis rats was specific for the colon and was not seen at the DRG level, although we did find evidence of reduced csk mRNA expression in the DRGs L6-S1 (Table 3).

**Table 3:** mRNA expression of P2X3, cdk5, csk and CASK in the colon and DRGs (Th13-L2 and L6-S1).

Tissue	Target gene	Control	Acute colitis	Post-colitis
		(n = 7)	(n = 5)	(n = 7)
Colon	P2X_3_	1.06 ± 0.16	1.12 ± 0.34	0.91 ± 0.09
	cdk5	1.07 ± 0.16	0.86 ± 0.18	1.54 ± 0.16 ^#^
	csk	1.34 ± 0.38	0.48 ± 0.13	3.52 ± 0.60 * ^#^
	CASK	1.12 ± 0.20	1.10 ± 0.17	1.83 ± 0.21 ^#^
Th13-L2	P2X_3_	0.95 ± 0.05	1.06 ± 0.12	1.08 ± 0.04
	cdk5	1.29 ± 0.24	1.60 ± 0.12	1.69 ± 0.56
	csk	1.11 ± 0.22	0.86 ± 0.08	0.77 ± 0.22
	CASK	1.38 ± 0.26	1.56 ± 0.30	2.22 ± 1.18
L6-S1	P2X_3_	1.08 ± 0.09	0.86 ± 0.05	0.98 ± 0.05
	cdk5	1.04 ± 0.12	0.79 ± 0.10	0.81 ± 0.15
	csk	1.05 ± 0.14	0.83 ± 0.11	0.38 ± 0.08 * ^#^
	CASK	1.07 ± 0.18	0.97 ± 0.09	1.14 ± 0.78

Data presented as mean ± sem for n = 5–10 animals per group. One-way ANOVA followed by SNK.

* p<0.05, significantly different from control

# p<0.05, significantly different from acute-colitis.

**Table 4:** Protein expression of CGRP and P2X_3_ in the dorsal root ganglia.

Staining	DRG level	Control	Acute colitis	Post-colitis
*Number of positive neurons per mm^2^*				
CGRP	Th13-L2	47 ± 17	59 ± 25	74 ± 33
	L6-S1	32 ± 15	28 ± 11	35 ± 8
P2X_3_	Th13-L2	29 ± 6	38 ± 18	55 ± 21
	L6-S1	30 ± 17	16 ± 5	32 ± 6
*% of co-expressing neurons*				
P2X_3_ /CGRP co-expression	Th13-L2	9 ± 3%	4 ± 1%	6 ± 2%
	L6-S1	6 ± 1%	8 ± 3%	11 ± 4%
CGRP/P2X_3_ co-expression	Th13-L2	13 ± 4%	7 ± 1%	8 ± 3%
	L6-S1	7 ± 2%	14 ± 4%	12 ± 4%

Data presented as mean ± sem for n = 3 animals per group and 3 DRGs per animal for each level. DRGs, dorsal root ganglia. Two-way ANOVA, no significant effects.

## Discussion

This study was designed to evaluate P2X_3_ receptor contribution to visceral mechanosensitivity. Our results indicate that P2X_3_-mediated signaling is not involved in colonic mechanosensitivity under physiological conditions, but contributes to a different extent to visceral hypersensitivity during acute TNBS-colitis and in the post-inflammatory phase.

### P2X_3_ receptors do not contribute to colonic mechanosensitivity under physiological conditions

In our experiments, A-317491 did not affect VMRs in control rats. A-317491 is a potent and selective antagonist with nanomolar affinity for P2X_3_ units [20]. A-317491 shows very poor central nervous system penetration and can thus be considered a peripherally acting blocker [28,29]. Therefore, our results suggest that at least peripheral P2X_3_ receptors do not contribute to colonic mechanosensitivity under physiological conditions, which is in line with previous evidence [14,20]. Shinoda et al. [14] reported that pelvic afferent nerve discharge in response to colorectal distension remained unaffected in P2X_3_^-/-^ mice. In the same study, VMRs in P2X_3_^-/-^ mice were significantly attenuated whereas ATP release to colorectal distension remained unaltered, leading the authors to conclude that under physiological conditions only central, but not peripheral P2X_3_ receptors contribute to colonic mechanosensitivity [14]. Such a central mechanism of action could be located at the level of the spinal cord where presynaptic P2X_3_ channels facilitate the release of excitatory glutamate [11]. The lack of a peripheral contribution of P2X_3_ to sensory signaling under physiological conditions is further suggested by the finding that double P2X_2_^-/-^ P2X_3_^-/-^ mice show similar increases in jejunal afferent nerve firing in response to gut distension compared to controls [13]. Likewise, the P2X_1_/P2X_3_ receptor antagonist TNP-ATP, which is also devoid of central nervous system penetration [30], did not affect *in vivo* nociceptive behavior to colorectal distension in rats under control conditions [18]. However in contrast, pelvic afferent nerve discharge to gut distension was significantly attenuated by TNP-ATP in healthy control rats [16].

### P2X_3_ receptors contribute to visceral hypersensitivity during acute TNBS-colitis and in the post-inflammatory phase

Our *in vivo* data of increased purinergic signaling via P2X_3_ channels during acute TNBS-colitis are in line with previous *in vitro* findings of Wynn et al. [17] who showed that pelvic afferent nerve firing in response to colorectal distension was significantly increased in an isolated colon segment from rats with acute TNBS colitis and that this increase could be attenuated by the P2X receptor antagonist PPADS and the P2X_1_/P2X_3_ receptor antagonist TNP-ATP. Our study elaborates on these results and now provides evidence from an *in vivo* set-up demonstrating that the selective P2X_3_ antagonist A-317491 dose-dependently, though not fully, reversed the increased VMRs to colorectal distension in rats with acute TNBS-colitis. This suggests that receptors containing the P2X_3_ unit contribute to visceral hypersensitivity during acute colitis. This antinociceptive effect of the P2X_3_ receptor antagonist was not mediated by changes in colonic compliance. This is important considering other antagonists such as TNP-ATP and PPADS also block P2X_1_ receptors expressed on smooth muscle cells in the rat distal colon [31]. Moreover, P2X_3_ receptors on myenteric neurons contribute to the initiation of reflex contractions when the intestinal pressure rises and could therefore modulate colonic compliance [32].

In a rat model for non-inflammatory visceral pain, induced by intraperitoneal injection of acetic acid, A-317491 has previously been shown to reduce nociception with an IC_50_ of 27 μmol/kg, which is approximately 15 mg/kg [20]. On the other hand, A-317491 had no effect on VMRs in a zymosan-induced model of acute colonic inflammatory hypersensitivity, even after administration of 100 μmol/kg (approximately 56.6 mg/kg) [20]. We can only speculate on the reasons underlying the discrepancy in the results between the latter study and our own findings. Firstly, the difference in experimental models for visceral hypersensitivity should be considered (TNBS *vs* zymosan). Secondly, in the zymosan-study, Sprague-Dawley rats were subjected to 3 colorectal distension protocols: the first before the induction, the second during acute colitis and the third 30 min after the second distension. In our hands, repeating distension protocols after a 30 min interval in male Sprague-Dawley rats induced hypersensitivity in control rats (data not shown), which might relate to differences in the distension protocol (6 distensions ranging from 10 to 80 mmHg *vs* three 60mmHg distensions). Therefore, only one distension protocol was performed in each rat in our set-up. In support of our results, A-317491 also exerted antinociceptive effects in models of somatic and neuropathic pain [20,29,33–35].

We additionally studied the effect of blockade of P2X_3_ receptor units in a rat model for post-inflammatory visceral hypersensitivity. In this model, we confirmed the resolution of TNBS-colitis by colonoscopic, macroscopic and microscopic evaluation of the colonic tissue, in addition to MPO activity. Despite the absence of inflammation, the VMRs to colorectal distension were increased in post-colitis rats. These increased VMRs were dose-dependently reduced and even normalized by A-317491. This strongly suggests an important role for P2X_3_ receptor units in post-inflammatory visceral hypersensitivity. A contribution of purinergic P2X receptors to post-infectious visceral hypersensitivity was previously suggested by Rong et al. [13] who demonstrated that the P2X receptor antagonist PPADS reduced increased jejunal afferent firing after *Trichinella spiralis* infection. Yet the exact P2X receptor subtype involved remained to be identified. Our study now provides evidence of enhanced P2X_3_ unit-mediated signaling in the post-inflammatory phase.

Evidence for a role for purinergic modulation of nociceptive responses, most likely mediated by P2X_3_ receptor units, has also been reported in other animal models of visceral hypersensitivity. Xu et al. [18] instilled acetic acid in the colon of neonate rats, which resulted in visceral hypersensitivity at the adult age; TNP-ATP reduced these increased VMRs. In addition, the intracolonic instillation of zymosan in control mice induced visceral hypersensitivity in the absence of overt colonic inflammation, while VMRs remained unaffected by zymosan exposure in P2X_3_^-/-^ mice [14]. Therefore, it seems that peripheral P2X_3_ receptor units contribute to visceral hypersensitivity, not only during and after the resolution of TNBS-colitis but also in other types of chemically-induced colonic hypersensitivity.

Another novel finding in this study is that the potency of A-317491 to reduce visceral hypersensitivity was different between acute colitis and the post-inflammatory phase. Visceral hypersensitivity during the acute inflammation was attenuated but not fully reversed by 25 mg/kg A-317491, whereas 10 mg/kg A-317491 almost completely normalized the increased visceral pain in the post-inflammatory phase. The difference in percentage of improvement between acute colitis and the post-inflammatory phase was statistically significant for both 10 and 25 mg/kg of A-317491. Of note, by expressing the degree of improvement as a percentage, we corrected for the tendency of higher VMRs in vehicle-treated rats during acute TNBS-colitis compared to vehicle-treated post-colitis rats.

Several mechanisms could underlie the difference in potency of A-317491 in reducing visceral hypersensitivity between the acute phase of colitis and the post-inflammatory phase. Firstly, there was a significant increase in distension-induced release of ATP, the endogenous ligand for P2X_3_ receptors, during acute colitis whereas ATP release had returned to control values in the post-inflammatory condition. ATP is present in the cell in millimolar concentrations and during inflammation, extracellular ATP levels increase due to active release as well as passive leakage from damaged or dying cells, in combination with a downregulation of ATP breakdown [36]. Being a competitive antagonist for the P2X_3_ unit, A-317491 can be displaced from the binding site by excess ATP [20]. In addition, at the level of the primary sensory afferent, ATP potentiates the effect of other known nociceptive mediators such as protons, capsaicin and 5-hydroxytryptamine [37]. Therefore the increased availability of extracellular ATP during acute TNBS-colitis, most likely contributes to the enhanced P2X_3_-mediated signaling during the acute inflammatory phase. Secondly, differences in the P2X_3_ receptor expression in the acute versus the post-inflammatory phase may also contribute to the difference in A-317491 potency. However, we found no evidence of altered P2X_3_ expression at the mNRA or protein level in the colon or DRGs, neither during acute colitis nor in the post-inflammatory phase, arguing against a significant increase in P2X_3_ receptors in our study. In addition, we could not demonstrate enhanced colocalization between P2X_3_ and CGRP either. In contrast, enhanced colocalization during acute TNBS-colitis as was previously reported by Wynn *et al*. [17]. Besides increased mediator release or increased receptor expression, sensitization of P2X_3_ receptors may provide a plausible explanation for these results. The signaling of extracellular ATP binding to P2X_3_ receptor is transduced in the intracellular environment by a series of adaptor and scaffold molecules among which are kinases such as Cdk5, Csk, and CASK. Cdk5 and Csk modulate the electrical properties of P2X_3_ receptors whereas CASK interacts with the P2X_3_ receptor to positively enforce the receptor’s stability and functional responsiveness [38].

To investigate their involvement, we performed RT-PCR for Cdk5, Csk and CASK mRNA expression in the colon and DRGs (Th13-L2 and L6-S1) of control, acute colitis and post-colitis rats. We found that in the post-colitis group, colonic mRNA expression of all three targets was significantly increased, whereas mRNA levels were similar in acute colitis and controls. The increased expression in the post-colitis group was specific for the colon and was not seen at the DRG level. These findings point towards P2X_3_ receptor sensitization in the post-colitis phase, that may underlie—or at least contribute to—the persistent post-inflammatory visceral hypersensitivity in our study.

The observation of an increased potency of A-317491 to reduce hypersensitivity in the post-colitis phase compared to the acute inflammatory event is an interesting observation that warrants further study. In fact few studies compare mediator contribution to hypersensitivity in an acute versus a post-inflammatory setting. Similar evidence was presented for acid sensing ion channels type 3 (ASIC3), as these were upregulated during active Crohn’s disease and contributed to peripheral sensitization by inflammatory mediators but were not involved in non-inflammatory visceral hypersensitivity according to another group [39–41]. To further investigate whether this increased potency in the post-inflammatory phase is specific for P2X_3_ receptor mediated signaling, we conversely compared the antinociceptive effect of a selective histamine H_1_ receptor antagonist levocetirizine in the acute and post-inflammatory setting (S1 Fig). We recently demonstrated that levocetirizine fully reversed post-inflammatory visceral hypersensitivity in the post-inflammatory phase at a dose of 1 mg/kg [21]. Similar to our results for A-317491, levocetirizine was more effective in reducing hypersensitivity in the post-inflammatory phase compared to the effect during acute TNBS-colitis (S1 Fig). As H_1_ receptors contribute to afferent nerve signaling in the gut wall [42], this suggests a more general upregulation of visceral afferent sensitivity in the post-inflammatory phase of TNBS-colitis.

In summary, we evaluated the antinociceptive effects of A-317491, a selective P2X_3_-unit antagonist, on visceral pain. A-317491 dose-dependently reduced visceral hypersensitivity during acute TNBS-colitis and fully abolished increased VMRs after resolution of colitis without affecting visceral sensitivity in controls. The antinociceptive effect of A-317491 was more pronounced in the post-inflammatory phase of colitis compared to the acute inflammatory phase, most likely due to displacement of A-317491 by excess ATP release during acute TNBS-colitis and sensitization of P2X_3_ receptors in the post-inflammatory phase of colitis.

Hence, purinergic P2X_3_ receptor units do not seem to be involved in sensory signaling under physiological conditions but mediate visceral hypersensitivity with a different potency during acute TNBS-colitis and in the post-inflammatory phase. These findings validate P2X_3_ receptors as a potential new target in the treatment of abdominal pain syndromes such as IBS and IBD.

## Supporting Information

S1 Fig Levocetirizine in acute TNBS-colitis compared to the post-inflammatory phase of colitis. Data are expressed as the percentage of improvement of normalization: 0% means no improvement and thus the same level of hypersensitivity as vehicle-treated rats during acute TNBS-colitis or in the post-inflammatory phase, whereas 100% means complete normalization of the increased VMRs (reaching the level of vehicle-treated controls). Levocetirizine (1 mg/kg) more potently reduced visceral hypersensitivity in the post-inflammatory phase of colitis compared to the acute inflammatory phase of colitis. Generalized estimating equations, LSD post-hoc test, n = 5–8; * p<0.05, significantly different compared to acute colitis.
(TIF)
