# Supplementary figures and images for: P2X3 Receptors Mediate Visceral Hypersensitivity during Acute Chemically-Induced Colitis and in the Post-Inflammatory Phase via Different Mechanisms of Sensitization
Source: PLoS One. 2015 Apr 17;10(4):e0123810. doi: 10.1371/journal.pone.0123810 (PMC4401691; doi:10.1371/journal.pone.0123810)

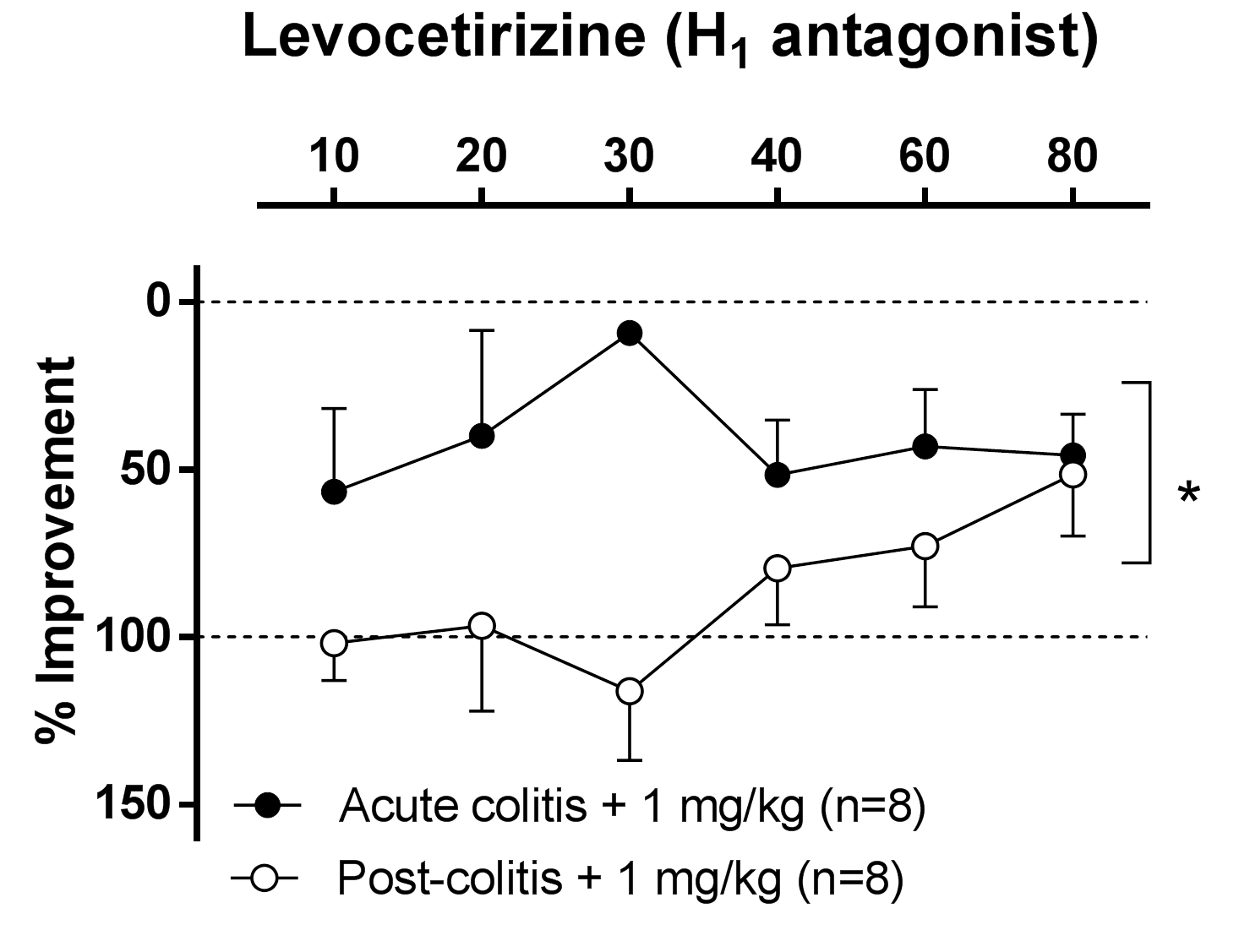

Supplement: S1 Fig — Data are expressed as the percentage of improvement of normalization: 0% means no improvement and thus the same level of hypersensitivity as vehicle-treated rats during acute TNBS-colitis or in the post-inflammatory phase, whereas 100% means complete normalization of the increased VMRs (reaching the level of vehicle-treated controls). Levocetirizine (1 mg/kg) more potently reduced visceral hypersensitivity in the post-inflammatory phase of colitis compared to the acute inflammatory phase of colitis. Generalized estimating equations, LSD post-hoc test, n = 5–8; * p<0.05, significantly different compared to acute colitis. (TIF) [file pone.0123810.s001.tif]
